# Assessment of DNA Topoisomerase I Unwinding Activity, Radical Scavenging Capacity, and Inhibition of Breast Cancer Cell Viability of *N*-alkyl-acridones and *N*,*N′*-dialkyl-9,9′-biacridylidenes

**DOI:** 10.3390/biom9050177

**Published:** 2019-05-08

**Authors:** Marios G. Krokidis, Zara Molphy, Eleni K. Efthimiadou, Marianna Kokoli, Smaragda-Maria Argyri, Irini Dousi, Annalisa Masi, Kyriakos Papadopoulos, Andrew Kellett, Chryssostomos Chatgilialoglu

**Affiliations:** 1 Institute of Nanoscience and Nanotechnology, N.C.S.R. “Demokritos”, 15310 Agia Paraskevi-Athens, Greece; m.krokidis@inn.demokritos.gr (M.G.K.); efthim@chem.uoa.gr (E.K.E.); marianna_kokoli@yahoo.gr (M.K.); argyri@student.chalmers.se (S.-M.A.); i.dousi@inn.demokritos.gr (I.D.); 2 School of Chemical Sciences and National Institute for Cellular Biotechnology, Dublin City University, Glasnevin, 9 Dublin, Ireland; zara.molphy2@mail.dcu.ie (Z.M.); andrew.kellett@dcu.ie (A.K.); 3 Department of Chemistry, National and Kapodistrian University of Athens, 15784 Zografou, Greece; 4 ISOF, Consiglio Nazionale delle Ricerche, Via Piero Gobetti 101, 40129 Bologna, Italy; annalisa.masi@isof.cnr.it

**Keywords:** *N*,*N′*-dialkyl-9,9′-biacridylidenes, topoisomerase I, DNA intercalation, DNA binding, radical scavenging capacity, cytotoxic activity

## Abstract

The anticancer activity of acridone derivatives has attracted increasing interest, therefore, a variety of substituted analogs belonging to this family have been developed and evaluated for their anti-cancer properties. A series of *N*-alkyl-acridones **1**–**6** and *N*,*N*′-dialkyl-9,9′-biacridylidenes **7**–**12** with variable alkyl chains were examined for their topoisomerase I activity at neutral and acidic conditions as well as for their binding capacity to calf thymus and possible radical trapping antioxidant activity. It was found that at a neutral pH, topoisomerase I activity of both classes of compounds was similar, while under acidic conditions, enhanced intercalation was observed. *N*-alkyl-acridone derivatives **1**–**6** exhibited stronger, dose-dependent, cytotoxic activity against MCF-7 human breast epithelial cancer cells than *N*,*N′*-dialkyl-9,9′-biacridylidenes **7**–**12**, revealing that conjugation of the heteroaromatic system plays a significant role on the effective distribution of the compound in the intracellular environment. Cellular investigation of long alkyl derivatives against cell migration exhibited 40–50% wound healing effects and cytoplasm diffusion, while compounds with shorter alkyl chains were accumulated both in the nucleus and cytoplasm. All *N*,*N′*-dialkyl-9,9′-biacridylidenes showed unexpected high scavenging activity towards DPPH or ABTS radicals which may be explained by higher stabilization of radical cations by the extended conjugation of heteroaromatic ring system.

## 1. Introduction

Acridone derivatives have been applied in clinics as anti-tumor chemotherapeutics since the 1970s. The cytotoxicity of acridone and acridine-based compounds arise not only from their direct binding to DNA but also from interactions with specific biological targets [1]. The unique planar ring structure of these derivatives may allow them to act either as topoisomerase poisons or as G-quadruplexes-DNA stabilizing agents. Additional telomerase and protein-kinase inhibitory activity may also contribute to cytotoxic effects in cancer treatment [2]. Analogs with a 2,2′-bis-pyridyl group substituted on the acridone scaffold or with a tetracyclic acridone structures, named as zanthacridones, showed broad antitumor activity against numerous cancer cell lines [3]. Derivatives with terminal ammonium substituents at C2 and C7 positions on the acridone moiety showed significant activity against leukemia CCRF-CEM cells along with limited toxic effect against normal cells proliferation [4]. These well-recognized properties of acridones are attributed to their semi-planar heterocyclic structures as well as N-substitution, which facilitates unique interactions with different molecular targets. For example, in a study by Xia et al., using MCF-7 xenograft mouse model, it was indicated that acridone could induce cell apoptosis, inhibiting breast cancer resistance protein (ABCG2), indicating strong in vivo potential [1]. A series of 2-fluoro N-substituted acridones with varying alkyl side chain length have been reported, exhibiting promising cytotoxic effects against sensitive and resistant cancer cell lines while their DNA-binding properties, based on their affinity or intercalation with CT-DNA, were evaluated [5]. Among them, a tricyclic N-substituted acridone with a fluorine group at the C-2 position additional to a secondary amine side chain containing a tertiary amino group was responsible for marked cytotoxic activity [5]. A series of substituted 9-aminoacridines were screened against MiaPaCa-2, SU86.86, and BXPC-3 pancreatic cancer cells, presenting low micromolar antiproliferative properties in the three distinct cell lines. Here, the resulting activity was derived from the induction of G1-S phase arrest, as the drug–DNA complex formed blocked topoisomerase II binding activity, leading to catalytic inhibition of the enzyme and the induction of apoptosis or programmed cell death [6]. In recent work by Zhang et al., a series of novel pyridyl-derived acridones were synthesized by combining a pyridyl moiety and the acridone-4-carboxamide scaffold. The majority of these compounds possessed cytotoxicity against K562 cells or inhibition of topoisomerase I activity, and induced apoptosis through mitochondrial pathway dysregulation [2].

DNA supercoiling is generated by metabolism and typically tends to be overwound (positively supercoiled) upstream of replication or transcription forks and underwound (negatively supercoiled) downstream of these forks [7]. Topological constraints can be relieved enzymatically with DNase I or DNA topoisomerases. DNA topoisomerases (topo) are ubiquitous enzymes that maintain cellular function by modulating the topology of supercoiled DNA during processes including gene transcription, DNA replication, recombination, and repair and, therefore, represent an attractive drug target [8]. The enzyme first acts by cleaving one (topo-I) or two (topo-II) strands of DNA through a transesterification reaction with active site tyrosine acting as the nucleophile that attacks the DNA phosphodiester backbone. This results in the generation of a transient break, which permits topological changes to occur within the cell prior to strand resealing. In recent years, many planar polycyclic organic molecules have been identified to alter topo enzyme activity through catalytic inhibition (camptothecin and derivatives of it) or poisoning (doxorubicin) [9,10]. Classical models for the reversible binding of drug molecules to duplex DNA include (i) groove binding (major and minor), (ii) intercalation, and (iii) insertion. Since these binding modes, in particular intercalation by planar heterocycles, affect supercoiling, valuable binding information can be identified in the presence of topoisomerase enzymes.

**Figure 1:**
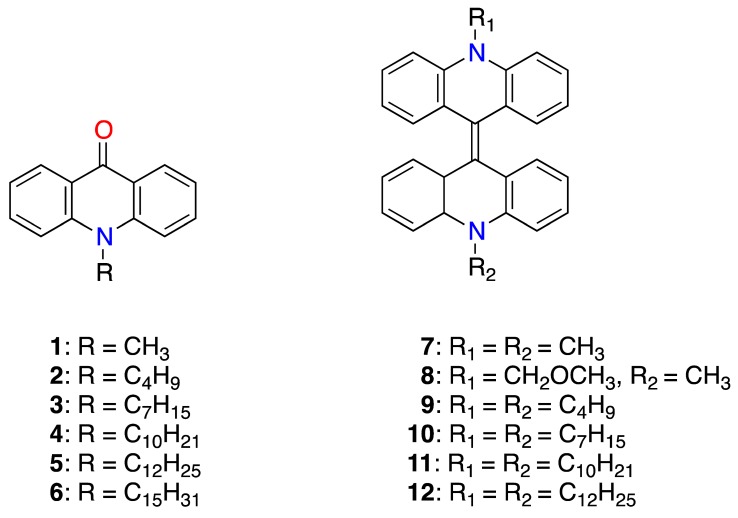
Chemical structures of *N*-alkyl-acridones 1–6 and *N*,*N′*-dialkyl-9,9′-biacridylidenes **7**–**12**.

In the present work, we examined the topoisomerase I, DNA binding, cytotoxic effects, as well as the radical trapping antioxidant activity of a series of *N*-alkyl-acridones and *N*,*N′*-dialkyl-9,9′-biacridylidenes with short and long alkyl chains substitution (Figure 1). *N*,*N′*-dialkyl-biacridylidenes **7**–**12** were synthesized from the corresponding *N*-alkylacridones **1**–**6** by reductive coupling with zinc powder in dry ethanolic hydrogen chloride solution or from lucigenin in alkaline methanolic solution (derivative **8**), while *N*-alkylacridones **1**–**6** from acridone and the corresponding alkyl bromides under phase-transfer conditions [11,12,13]. Optical and confocal microscopy imaging was assessed to clarify the role of alkyl side chain substitution in the intracellular localization of cytotoxic derivatives. 

## 2. Materials and Methods 

### 2.1. Materials, Chemical Compounds and Cell Cultures Conditions

HPLC-grade solvents were purchased from Merck (Steinheim, Germany) and Fisher Scientific (Waltham, MA, USA). Ultrapure water (18.3 MΩcm), distilled, and deionized water (Milli-Q water) were purified using a Milli-Q system (Merck-Millipore, Bedford, USA). High glucose Dulbecco’s modified Eagle Medium (DMEM) was purchased from Sigma (St. Louis, MO, USA). Trypsin-EDTA, L-glutamine, penicillin–streptomycin solution, and heat inactivated fetal bovine serum (FBS) were obtained from Biochrom KG, (Berlin, Germany). Human Caucasian breast adenocarcinoma cell line (MCF-7) was obtained from the American Type Culture Collection (ATCC; Manassas, VA, USA) and cultured as monolayers in complete medium Dulbecco’s Modified Eagle’s Medium (DMEM, Sigma, Steinheim, Germany) at 37 °C in an atmosphere of 5% (*v*/*v*) CO_2_ and 95% at relative humidity. Cells were seeded in 75 cm^2^ plastic tissue culture flasks and cultured in DMEM supplemented with 10% FBS, 2 mM l-glutamine, 100 U/mL penicillin, and 100 μg/mL streptomycin, washed with phosphate buffered saline (PBS) and harvested by trypsinization with 0.05% (*w*/*v*) trypsin in PBS containing 0.02% (*w*/*v*) EDTA (Sigma, St. Louis, MO, USA). 

### 2.2. Synthetic Procedures for the Preparation of N-Alkylacridones **1**–**6** and N,N′-Dialkyl-9,9′-Biacridylidenes **7**–**12**

*Synthesis of N-alkylacridones* (**1**–**6**), 30 mmol of the corresponding n-alkylbromide (methyl iodide for **1**) were added under stirring to a mixture containing 3.9 g acridone (20 mmol), 20 mL aqueous sodium hydroxide solution (50%), 40 mL toluene, and 2.0 g tetrabutylammonium bromide (6.2 mmol) under reflux for ca. 10 h. After cooling, the reaction mixture was then worked up with ether and water and the combined organic extracts were evaporated under reduced pressure (rot-evaporator). The resulting yellow to orange colored products were collected and purified by crystallization from ethanol or ether-petroleum ether. Chemical yields, 56 to 80%. **1**: m.p. 201 °C; ^1^H NMR (CDC1_3_, 250 MHz): *δ* (ppm) = 8.52 (d, *J* = 8.4 Hz, 2H), 7.65 (m, 2H), 7.43 (d, *J* = 8.1 Hz, 2H), 7.25 (m, 2H), 3.84 (s, 3H); **2**: m.p. 100 °C; ^1^H NMR (CDC1_3_, 250 MHz): *δ* (ppm) = 8.5 (d, *J* = 8.4 Hz, 2H), 7.62 (m, 2H), 7.43 (d, *J* = 8.1 Hz, 2H), 7.25 (m, 2H), 7.25 (m, 4H), 4.24 (t, 2H, NCH_2_), 1.8 (m, 2H), 1.55 (m, 2H), 1.05 (t, 3H); **3**: m.p. 91 °C; ^1^H NMR (CDC1_3_, 250 MHz): *δ* (ppm) = 8.52 (d, *J* = 8.4 Hz, 2H), 7.64 (m, 2H), 7.44 (d, *J* = 8.1 Hz, 2H), 7.22 (m, 4H), 4.22 (t, 2H, NCH_2_), 1.83 (m, 2H), 1.44 (m, 8H), 0.92 (t, 3H); **4**: m.p. 88 °C; ^1^H NMR (CDC1_3_, 250 MHz): *δ* (ppm) = 8.52 (d, *J* = 8.4 Hz, 2H), 7.55 (m, 2H), 7.43 (d, *J* = 8.1 Hz, 2H), 7.24 (m, 4H), 4.23 (t, 2H, NCH_2_), 1.83 (m, 2H), 1.34 (m, 16H, 0.92 (t, 3H); **5**: m.p. 86 °C; ^1^H NMR (CDC1_3_, 250 MHz): *δ* (ppm) = 8.54 (d, *J* = 8.4 Hz, 2H), 7.62 (m, 2H), 7.44 (d, *J* = 8.1 Hz, 2H), 7.24 (m, 4H), 4.22 (t, 2H, NCH_2_), 1.82 (m, 2H), 1.3 (m, 20H, 0.94 (t, 3H); **6**: ^1^H NMR (CDC1_3_, 250 MHz): *δ* (ppm) = 8.52 (d, *J* = 8.4 Hz, 2H), 7.62 (m, 2H), 7.44 (d, *J* = 8.1 Hz, 2H), 7.24 (m, 4H), 4.22 (t, 2H, NCH_2_), 1.82 (m, 2H), 1.3 (m, 26H, 0.94 (t, 3H). 

*Synthesis of 9,9′-biacridylidenes* (**7**, **9**–**12**), to a stirred solution of 5.0 mmol *N*-alkylacridone and 2.28 g zinc powder (35 mmol) in absolute ethanol (20 mL) and under an inert atmosphere, dried saturated ethanolic hydrogen chloride solution (30 mL) was added dropwise while cooling with ice (0 °C). The cooling bath was then removed and the reaction continued until most of the zinc was consumed. The reaction was terminated by pouring the mixture into water. The crude product was filtered off and rinsed well with water. Compounds were purified by crystallization from toluene and the chemical yields of the crude products were in all cases 70 to 85%. The most typical signals appeared in ^1^H NMR (CDC1_3_, 250 MHz) were δ = 4.01 (pseudo-t, 4H, NCH_2_), 6.65 (m, 4H), 7.03 (m, 4H) and 7.23 (m, 8H). **7:** m.p. 385 °C; **9**: m.p. 252 °C; **10**: m.p. 156 °C; **11**: m.p. 129 °C; **12** m.p. 118 °C. *Synthesis of N-methoxymethyl-N′-methyl-9,9′-biacridylidene, **8:*** Lucigenin (500 mg 1.0 mmol) and 1g NaOH (25.0 mmol) were dissolved in 50 mL methanol and the mixture was stirred under reflux. After 2 h, the yellow precipitate was filtered off, washed with cold methanol, and dried under vacuum; chemical yield 266 mg (64%); m.p. 275 °C; ^1^H NMR (CDC1_3_, 250 MHz): *δ* (ppm) = 3.56 (s, 3H, NCH_3_), 3.61 (s, 3H, OCH_3_), 5.42 (s, 2H, NCH_2_O), 6.82 (m, 4H), 7.08 (m, 4H), 7.27 (m, 8H).

### 2.3. Topoisomerase-I Mediated DNA Relaxation Assay

This experimental procedure was adapted from a method previously published elsewhere [14]. Briefly, acridones **1**–**6** and biacridylidenes **7**–**12** were initially dissolved in DMSO and further diluted in either 80 mM HEPES (pH 7.2) or 80 mM phosphate buffer (pH 5.8). We exposed 400 ng of pUC19 plasmid DNA (NEB, N3041, Ipswich, MA, USA) to varying concentrations of drug (0.01–400 µM) for 30 min at room temperature in a final volume of 20 µL containing 80 mM HEPES (pH 7.2) or 80 mM phosphate buffer (pH 5.8), 10× CutSmart^®^ buffer (NEB, B7204), and 100× BSA (NEB, B9000). Added to the mixture, was 1 unit of topoisomerase I (E. coli) (NEB, M0301) and incubated for 20 min at 37 °C to ensure relaxation of the plasmid DNA. The reaction was stopped through the addition of SDS and protein kinase, to a final concentration of 0.25% and 250 µg/mL, respectively. To remove protein from the DNA, samples were then incubated for 30 min at 50 °C. DNA supercoiling was assessed by agarose gel electrophoresis. A 6× loading buffer (Fermentas, Waltham, MA, USA) was added and the reaction mixture was loaded onto a 1.2% agarose gel in the absence of ethidium bromide (EtBr). Topoisomers of DNA were separated by electrophoresis in 1× TBE buffer at room temperature for 180 min at 40 V followed by 150 min at 50 V. The agarose gel was post-stained using an EtBr bath and photographed using a UV trans-illuminator.

### 2.4. DNA Binding Studies

DNA-binding properties of analogues were investigated using UV–Vis absorbance spectroscopy. All UV–Vis spectra were recorded on a JASCO V-650 spectrophotometer. In UV titration experiments, the spectra of calf-thymus DNA (CT-DNA, Sigma, St. Louis, MO, USA) in the presence of the compound have been recorded for a constant CT-DNA concentration in diverse [complex]/[CT-DNA] mixing ratios (*r*). The intrinsic binding constant, K_b_, of the complex with CT DNA was determined through the UV spectra of the complex recorded for a constant complex concentration in the absence and presence of CT-DNA for diverse *r* values. Each experiment was repeated in triplicate. A solution of CT-DNA was prepared using 150 mM NaCl in 15 mM trisodium citrate at pH 7.0, followed by stirring for 48 h at 4 °C. A_260_/A_280_ ratio at 1.47 and A_260_/A_230_ ratio at 15.39 (≥2) indicated that CT-DNA was sufficiently free from organic substances and 82.5% pure of protein contamination [15,16]. The concentration of duplex DNA was determined following the Beer Lambert Law, with a molar extinction coefficient of 6600 M^−1^cm^−1^ and was calculated to 59.42 μM. Analogs were diluted in a tris-citrate buffer (containing 150 mM NaCl and 15 mM trisodium citrate at pH 7.0) in the presence of approximately 1.0% DMSO and incubation with CT-DNA was carried out for 24h. 

### 2.5. DPPH and ABTS Radical Scavenging Activity Assays 

The radical scavenging capacity of biacridylidenes **7**–**12** toward DPPH (1,1-diphenyl-2-picryl-hydrazyl, Sigma, Steinheim, Germany) was determined according to the method of Brand-William et al., [17] with some modifications. Briefly, 2.0 mL of 0.1 mM DPPH solution in ethanol was incubated with 2.0 mL of varying concentrations of compounds **7**–**12** (10 to 200 μM). The reaction mixture was stirred for 60 min at room temperature and the absorbance of the resulting solution was read at 517 nm against a blank. The radical scavenging activity was measured as a decrease in the absorbance of DPPH and was calculated using the following equation:

radical scavenging activity (%) = [(A_0_ − A)/A_0_] × 100 (1)

where: A_0_ represents the absorbance of DPPH in the absence of the analog and A the absorbance of DPPH in the presence of biacridylidene compound. The ABTS assay was performed according to known protocols with some modifications [18]. Initially, 2,2′-azino-bis-(3-ethylbenzothiazoline-6-sulfonic acid) diammonium salt (ABTS, Sigma, Steinheim, Germany) was dissolved in a mixture of water/ethanol (80:20) at a concentration of 7 mM and reacted with a 2.45 mM potassium persulfate solution overnight at room temperature. The absorbance of ABTS radical cation was measured at 734 nm in the absence and presence of the tested compounds (10–250 μΜ) upon 60 min incubation at room temperature. The IC_50_ values were defined as the amount of the radical scavenger necessary to decrease the initial DPPH or ABTS concentration by 50%. Trolox and caffeic acid were used as reference agents. All experiments were performed in triplicate. 

### 2.6. MTT Assay 

Cell viability was assessed in MCF-7 cells exposed to *N*-alkylacridones and *N*,*N′*-dialkyl-9,9′-biacridylidenes using the MTT assay [19]. Briefly, cells were seeded in 96-well flat bottom microplates (1.5 × 10^4^ cells in 0.1 mL of medium/well) in triplicate, allowed to grow overnight until 70% confluent in a controlled atmosphere (37 °C, 5% CO_2_ and 95% relative humidity), and then treated with a range of concentrations of test compounds. For dissolution and dilution of the compounds, 0.05–0.2% DMSO was utilized. After 24, 48, and 72 h of incubation, the medium was replaced with 0.1 mL of 3-(4,5-dimethylthiazol-2-yl)-2,5-diphenyl tetrazolium bromide (MTT, Sigma, Steinheim, Germany) solution (1 mg/mL diluted in DMEM) and the plate was incubated for another 4 h at 37 °C, 5% CO_2_. The dark-blue formazan crystals, which are precipitated by viable cells, were dissolved in DMSO. Results are expressed as a percentage (%) of growth with respect to control cells, using data from three independent experiments, measured at 540 nm (reference filter 620 nm) using a microplate reader (Sirio S, SEAC Radim group). 

### 2.7. Wound Healing Assay

A wound healing assay was used to assess cell migration after treatment with test compounds [20]. MCF-7 cells were seeded in six-well plates (1 × 10^6^ cells per well) and wounds were created in 90% confluent cells using a sterile pipette tip. Afterwards, cells were washed with phosphate buffered saline (PBS) to eliminate free-floating cells, medium was added, and the culture plates were incubated at 37 °C. Experiments were conducted in triplicate. Wound healing was observed at 0 h, 24 h, and 48 h post-scratching, and representative images were obtained by a transmitted-light microscope equipped for live-cell imaging (OMAX Trinocular Compound EPI-Fluorescence Microscope M834FLR model on a ×4 magnification). The wound area percentage was calculated as the wound area from t = 24 h or t = 48 h vs. the wound area from t = 0 h in each group.

### 2.8. Confocal Microscopy Imaging and Localization Study

Cells (5 × 10^6^ cells/well) were grown on 0.22 cm^2^ glass cover slips (Fisher Scientific, Waltham, MA, USA) placed into six-well cultured plates containing 1.5 mL of culture medium for 24 h at 37 °C. The incubation of cells exposed to test analogs was for 3 h and 24 h at 37 °C. The cover slips were then recovered, rinsed twice with PBS, fixed with 10% paraformaldehyde in PBS, and nuclei stained with DAPI (Thermo Fisher Scientific, Waltham, MA, USA), 1% *w*/*v* in PBS. Subsequently, cover slips were washed three times with PBS and analyzed for imaging using a confocal laser scanning microscope ×60 magnifications (Leica TCS SP8 MP, inverted confocal microscope with Acousto-Optical Beam Splitter, Wetzlar, Germany). Images were acquired with the spectral detector of the microscope using appropriate emission wavelength ranges; DAPI stained nuclei were excited at 780 nm with a two-photon IR Mai Tai DeepSee Ti: Sapphire laser (Spectra-Physics/Newport, Santa Clara, CA, USA) and emission was recorded between 420–500 nm. Cellular fluorescence was analyzed using the Leica Application Suite software; LAS X software platform. 

### 2.9. Statistical Analysis

The data are presented as the mean ± standard deviation of the experiments performed in triplicate. Statistical comparisons were carried out using Student’s t test. *p* < 0.05 and *p* < 0.01 were considered to be significant.

## 3. Results and Discussion

### 3.1. Topoisomerase-I Mediated DNA Relaxation Assay

Interactions between both DNA and a drug molecule are dependent on their respective structural features, and interactions can be determined through several biochemical and biophysical experimental methods [21]. To characterize the intercalative ability of acridone and biacrylidene derivatives, topoisomerase-I (topo-I) mediated relaxation of negatively supercoiled DNA was examined. Ethidium bromide (EtBr) is routinely used as a reference compound to evidence topoisomerase I-induced DNA relaxation, while doxorubicin is employed a topoisomerase poison [22]. At low concentrations of EtBr exposure, the intercalation of the planar cationic molecule into a supercoiled plasmid induces complete relaxation of negatively supercoiled (SC) DNA resulting in a slower DNA migration pattern through the agarose medium (0.5 µM). As the concentration of the molecule is further increased, the DNA molecules wind in the opposite direction, inducing positively SC DNA with an electrophoretic mobility close to that of negatively charged SC DNA (1–10 µM) [23]. The unwinding profile observed in the case of EtBr is signature of classical intercalation by the planar heteroaromatic molecule. The concentration of a drug molecule required to produce fully relaxed DNA varies from compound to compound, reflecting their respective binding affinities to negatively SC DNA. The family of *N*-alkylacridones and *N*,*N′*-dialkyl-9,9′-biacrylidenes were investigated at both neutral (pH 7.2) and acidic (pH 5.8) environments and directly compared to control agents EtBr, doxorubicin, and simple unsubstituted acridone. 

**Figure 2:**
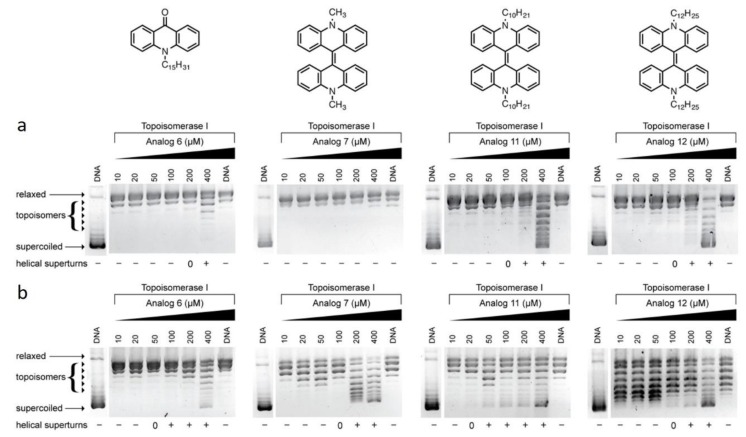
Topoisomerase-I mediated DNA relaxation assay in the presence of increasing concentrations (10–400 μΜ) of *N*-alkylacridone **6** and *N*,*N′*-dialkyl-9,9′-biacridylidenes **7, 11,** and **12** at pH 7.2 (**a**) and pH 5.8 (**b**), respectively.

At a neutral pH, acridone derivatives **2**–**5** have no influence on DNA supercoiling in the presence of topo-I (Figure S1). When the hydrocarbon tail of the *N*-alkyl-acridones increases in length from 12 to 15 carbon atoms (**6**) some evidence of unwinding was evident at higher concentrations (Figure 2a). A similar effect can be observed in the case of the *N*,*N′*-dialkyl-9,9-biacridylidene series, when the carbon chain length increases stepwise from 1 to 12 carbon atoms (**7**, **9**, **10**, **11,** and **12**) as shown in Figure 2a and Figure S2). Under acidic conditions, a release of supercoiling from the plasmid can be observed for pentadecyl-acridone **6** (Figure 2b). Similarly, when the hydrocarbon tail of biacridylidene derivatives increases in length (analogues **7**, **11** and **12**) positively charged supercoils appear at higher loading concentrations (Figure 2b).

### 3.2. DNA Binding Study

**Figure 3:**
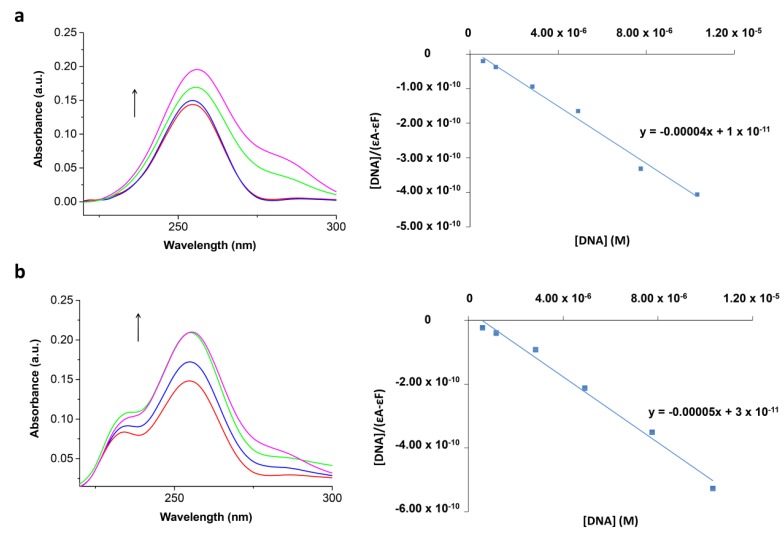
Absorption spectra of *N*-butylacridone **2** and *N*,*N′*-dibutyl-9,9′-biacridylidene **9** in 15 mM trisodium citrate-150 mM NaCl buffer (pH 7.0) in the presence of increasing amounts of CT-DNA after 24 h incubation. (**a**) R= [CT-DNA]/[acridone **2**] = 1/50, 1/10, 1/5, and 1/2 and the linear fit of [DNA]/(e_a_ − e_f_) vs. [DNA] for the titration of CT-DNA with acridone **2**; (**b**) R= [CT-DNA]/[biacridylidene **9**] = 1/50, 1/10, 1/5, and 1/2 and the linear fit of [DNA]/(e_a_ − e_f_) vs. [DNA] for the titration of CT-DNA with biacridylidene **9.**

Serial dilutions of each analog were incubated with CT-DNA. Titrations were performed by maintaining the analog concentration and varying [CT-DNA] by adding (*R* = [CT-DNA]/[Compound] = 1:50, 1:10, 1:5, 1:2). UV–Vis spectra of the samples incubated with CT-DNA were recorded at 37 °C for 24 h, indicating that a new complex formed between the compounds and the double-helix of CT-DNA. The intrinsic binding constant, *K*_b_ was calculated using the following equation:

[DNA]/(ε_a_ − ε_f_) = [DNA]/(ε_b_ − ε_f_) + 1/K_b_(ε_b_ − ε_f_) (2)

where *ε*_a_, *ε*_f_, and *ε*_b_ are the apparent, free, and bound complex extinction coefficients, respectively. *ε*_f_ was determined from the calibration curve of the analogues following Beer’s law, whereas *ε*_a_ is the ratio between the measured absorbance and the analogue concentration (*Absorbance*/compound concentration). The plot of [DNA]/(*ε*_a_ − *ε*_f_) versus [DNA] has a slope of 1/(*ε*_b_ − *ε*_f_) and the intercept is equal to 1/*K*_b_(*ε*_b_ − *ε*_f_); *K*_b_ is the ratio of the slope to the intercept [24]. In the intercalative binding mode, the π* orbital of the intercalated ligand can couple with the π orbital of the DNA base pairs, thus, decreasing the π→π* transition energy and resulting in bathochromism. Moreover, the coupling π orbital is partially filled by electrons, thus, decreasing the transition possibilities and concomitantly results in hypochromism. Generally, the binding of an intercalative molecule to DNA is accompanied by hypochromism and/or significant red-shift (bathochromism) in the absorption spectra due to the strong stacking interaction between the aromatic chromophore of the compound and DNA base pairs with the extent of hypochromism and red-shift commonly consistent with the strength of the intercalative interaction [25].The hyperchromic effect may be ascribed to external contact (electrostatic binding) or to partial unwinding of the helical structure of DNA, exposing more bases of DNA. Increasing concentrations of CT-DNA led to a modified absorption pattern in a selection of analogs from each family, as illustrated in Figure 3. Compounds were diluted in a mixture of DMSO (0.8% for **2**, and 1% for **9**) in tris-citrate buffer (containing 150 mM NaCl and 15 mM trisodium citrate at pH 7.0). Hyperchromicity was observed in *N*-butylacridone **2**, whereas hypochromicity was indicated at the *N*,*N′*-dibutyl-9,9′-biacridylidene **9**. The *K_b_* values were calculated (Table 1) and found to be similar to the reported data for the classical intercalator ethidium bromide, as its binding constant is in the order of 10^7^ M^−1^ [26]. The above results show the binding of *N*-alkylacridones and *N*,*N′*-dialkyl-9,9′-biacridylidenes to CT-DNA is likely to occur via intercalation. Binding properties of the above derivatives with CT-DNA were also investigated after 48 h incubation. The results, however, revealed that 24 h incubation is adequate for the compounds to bind CT-DNA. The binding constant (K_b_) in 24 h was also found to be stronger than that in 48 h (Table 1). 

### 3.3. DPPH and ABTS Radical Scavenging Activity 

Primary antioxidants are usually aromatic alcohols (phenols or polyphenols), which neutralize free radicals and other reactive oxygen species (ROS) via electron transfer or hydrogen atom transfer [26,27,28]. Similar behavior can be attributed, to a lesser extent, to secondary aromatic amines (anilines), which contain labile hydrogen atoms on the nitrogen atom. Studies on the antioxidant activity of diphenylamine derivatives showed that indeed the antioxidant effect of these derivatives lies in their NH functional group [29]. The reactivity of secondary amines, such as 9,10-dihydroacridine or unsubstituted 9,9′-dihydroacridine toward peroxyl radicals, was about five orders of magnitude lower than the corresponding value of the well-known antioxidant trolox. Kinetic measurements including various dihydroacridines revealed that the radical scavenging effect was essentially attributed to the N−H bond despite the fact that in 9,10-dihydroacridine the calculated C−H bond dissociation enthalpy at C9 is about 11 kcal/mol lower than the N−H [30]. The radical trapping activity of *N*,*N′*-unsubstituted or *N*,*N′*-substituted 9,9′-biacridylidenes, has to our knowledge not been explored. To our surprise, biacriylidenes **7**–**12** presented high radical scavenging activities towards DPPH and ABTS radicals, in some cases similar to that of trolox (Table 2). This behavior may be attributed to strong donating ability of labile electrons at N-atoms of the heteroaromatic system and stabilization of nitrogen or carbon radicals through the extended conjugation of the aromatic system. Their radical scavenging activities are expressed as IC_50_ or trolox-equivalent antioxidant capacity values (TEAC-values). TEAC values were calculated by dividing the mean value of IC_50_ of a compound through the IC_50_ of trolox. A lower TEAC value indicates a higher radical scavenging activity. 

### 3.4. MTT Assay

**Figure 4:**
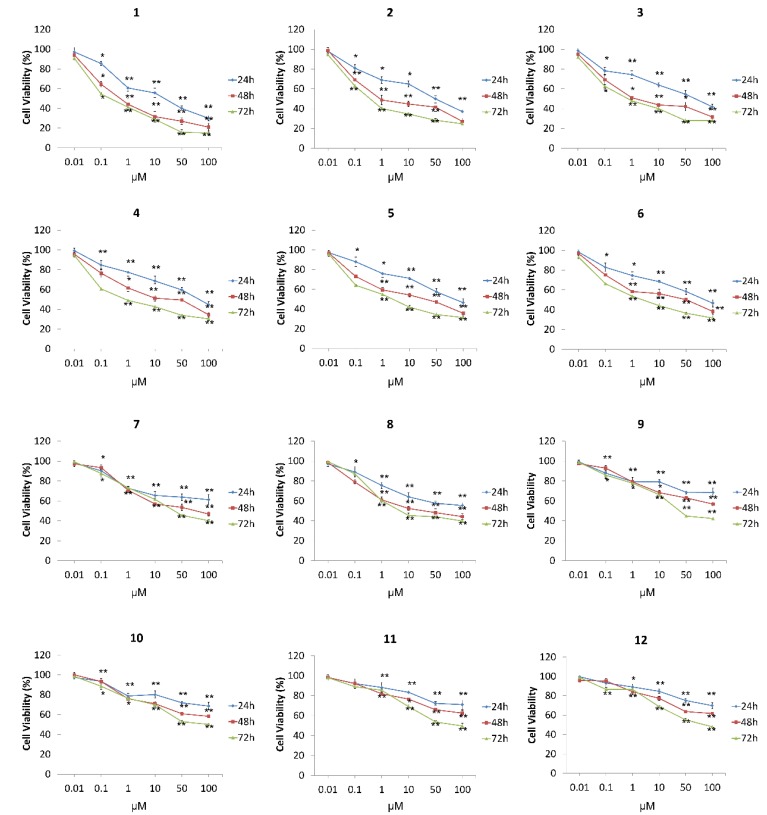
Dose-dependent response of MCF-7 epithelial breast cancer cells to test compounds **1**–**12**. The concentration range was from 0.01 to 100 μM and the incubation time was 24, 48, and 72 h in a serum containing medium. Under the same conditions, results are presented as percentage of growth in respect to the control cells. Each point represents the mean ± standard deviation from the experiments in triplicate. Asterisks mark the statistically significant levels using the Student t-test: * *p* < 0.05, ** *p* < 0.01, respectively, as compared to control cells.

This colorimetric assay is based on the reduction of the yellow water-soluble tetrazolium salt (MTT) into an insoluble crystalline blue formazan product by cellular oxidoreductases of viable cells. This transformation is not possible on dead cells, therefore, the resulting formazan crystal formation is proportional to the number of viable cells with an active metabolism. MTT assay was therefore employed to measure cell metabolic activity in MCF7 cells exposed to *N*-alkyl-acridones (**1**–**6**) and *N*,*N′*-dialkyl-9,9′-biacridylidenes (**7**–**12**). The compounds were found to clearly effect cell viability as concentration increased in the range of 0.01 and 100 μΜ, over 24, 48, and 72 h. As illustrated in Figure 4, *N*-alkylacridones showed significant activity in a dose-depended manner at 24 h. *N*-Methylaridone **1** exhibited the strongest effect with an IC_50_ value of 6.75 μΜ (Table 3), derivatives **2** and **3**, containing respective butyl and heptyl groups, followed thereafter (10.1 and 11.67 μΜ), while analogs **4**, **5**, and **6** had similar cytotoxic behavior (IC_50_ 13.80, 14.81 and 14.13 μΜ, respectively). On the other hand, *N*,*N′*-dialkyl-9,9′-biacridylidenes exhibited similar dose dependent activity, with attenuated viability status compared with the previous six acridone derivatives (Figure 4). Compounds **7** and **8** including methyl and methoxy-methyl groups in their scaffold were characterized by IC_50_ values 19.13 and 15.65 (Table 3) while **9**–**12** containing butyl, heptyl, decyl, and dodecyl alkyl chains, respectively, exhibited weaker cytotoxic effects with IC_50_ values at higher concentrations (28.40–33.95 μM, Table 3). Furthermore, all compounds were tested in a time-dependent manner of the doses applied. As presented in Figure 4, acridone derivatives **1** to **6** and biacridylidenes **7** to **12** present significant, dose-dependent inhibition of cell growth. Methyl acridone **1**, the most potent among the tested derivatives, pinpointed 100 μΜ as the maximum inhibitory effect on cell growth (20% at 48 h and 18% at 72h, respectively) compared to control cells (Figure 4). However, analogs **2**, at the same concentration, reduced viability rates significantly (approximately 75–65%) while biacridylidene analogs **7**–**12** presented inhibition rates of 58–38% at 48h and 40–52% at 72 h, respectively. 

### 3.5. Wound-Healing Assay

**Figure 5:**
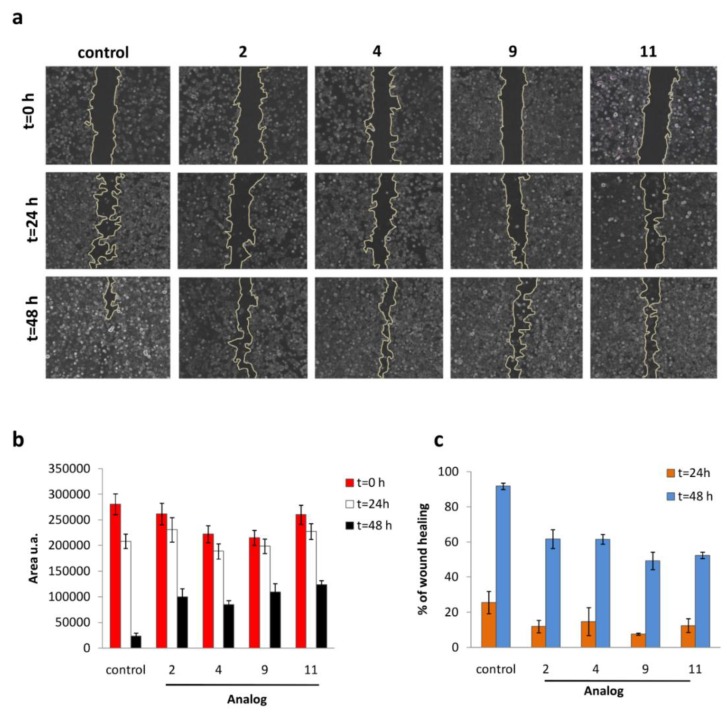
(**a**) Representative images show the scratch (wound) at t = 0, 24 and 48 h with/without drug treatments. Treatment with alkyl-acridones (**2** and **4**) and biacridylidenes (**9** and **11**) impairs migration of MCF-7 cells. Untreated cells efficiently restore the monolayer after 48 h. (**b**) Quantification of restored surface from three images for each timepoint in the absence/presence of analogs. (**c**) Graph illustrating the % wound healing in MCF-7 cells upon treatment with the compounds after 24 and 48 h. Data represent mean ± standard deviation for three different experiments.

The wound healing (scratch repair) assay represents a simple approach commonly used to measure basic cell migration parameters and cell–cell interactions mimicking to some extent in vivo conditions, identifying the degree of migration of cancer cells caused by treatments [31,32]. MCF-7 cells were seeded and allowed to reach 90% confluence. Cells were then wounded by scratching with a sterile pipette in the presence or absence of test derivatives and representative images were taken at 0, 24, and 48 h after the initial scratch was performed (Figure 5a). Control cells were healed after 48 while butyl-acridone **2** and heptyl-acridone **4** showed increased migration effectiveness compared to biacridylidenes **9** and **11** (60% vs. 50–55%, respectively, Figure 5c). 

### 3.6. Confocal Microscopy Imaging and Localization Study

**Figure 6:**
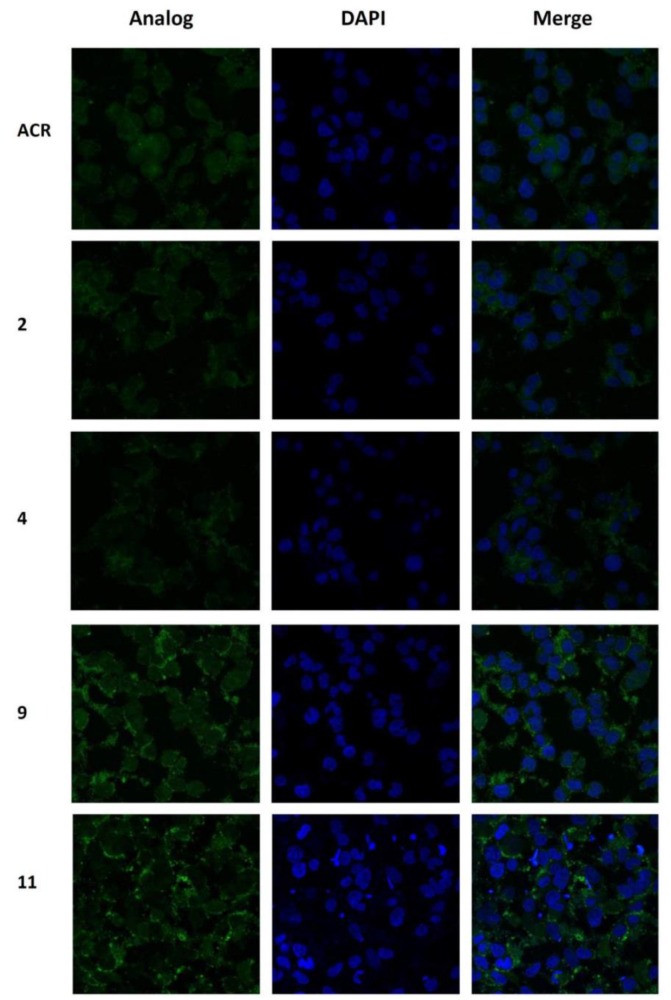
Determination of intracellular distribution of analogs **2**, **4**, **9,** and **11** in MCF-7 breast cancer cells. Acridone (ACR) was used as a control. Cells were treated with 1 μM of each derivative and after 3 h of incubation, cells were imaged by confocal microscopy. Blue is the DAPI (4′,6-diamidino-2-phenylindole) nuclear stain.

Both the substitution and the role of the carbon chain length on the intracellular localization of selected compounds were investigated using confocal microscopy. Control acridone (ACR) revealed a strong dose-dependent cytotoxic profile in MCF-7 cells with an IC_50_ value of 0.27 μΜ (Figure S3), while it presented a well-characterized localization in a greater extent in the cell nucleus and less in the cytoplasm as illustrated in Figure 6. MCF-7 cells were incubated with 1 μM of each analog for 3 h and the corresponding autofluorescence was depicted. Accumulation of **2** both in the nucleus and cytoplasm is shown in Figure 6**,** while analog **4**, which contains a longer alkyl chain, is mainly accumulated in the cytoplasm. The *N*,*N′*-biacridylidene **9** incorporating butyl as an alkyl chain allowed a small percentage of accumulation in the nucleus upon 3 h of incubation. On the other hand, derivative **11**, which includes a longer alkyl chain on the molecular scaffold, appeared to be diffused primarily in the cytoplasm (Figure 6). To further examine any correlation between the time of treatment and the resulting intracellular distribution, cells were treated with test derivatives for 24 h. Exposure to analog **9** and **11** caused no appreciable effect, revealing that a small percentage of the compound could penetrate the nucleus membrane. Analog **11** diffused into the cytoplasm while analog **9** still exhibited small nuclear localization with a greater accumulation of the compound remaining in the cytoplasm.

## 4. Conclusions

Acridone analogs are well known chemotherapeutic agents that can interact with nucleic acids. As such, structural modifications around this scaffold is of great interest, and facilitates the tuning of DNA intercalative properties [33]. The present study identified the topoisomerase I unwinding activity, DNA binding properties, and cytotoxic activity against MCF-7 breast cells of a series of *N*-alkylacridones and *N*,*N′*-dialkyl-9,9′-biacridylidenes substituted with varying alkyl chains. The topoisomerase-I mediated DNA relaxation assay can be successfully employed to determine the intercalating propensity of a drug molecule by observing the ability of topo-I to mediate relaxation of a topologically constrained negatively supercoiled DNA molecule. Our study revealed the promising potential mainly of the short alkyl substituted analogs after concentration dependent cytotoxicity screening on the selective cancer cell line. According to fluorescence microscopy, these compounds accumulated both in the nucleus and cytoplasm and potently inhibited cell proliferation instead of the longer substituted analogs which largely remained in the cytoplasm. These studies revealed that most alkyl-based acridone derivatives have potent cytotoxic activity compared with biacridylidenes with longer alkyl substituents. By interacting with DNA and inhibiting topoisomerases activity, biological activity was established for small planar polycyclic aromatic molecules, such as acridone, representing a promising lead moiety for the development of novel effective anticancer DNA binding agents.

## Figures and Tables

**Table 1:** K_b_ values of *N*-butylacridone **2** and *N*,*N′*-dibutyl-9,9′-biacridylidene **9** and the observed change in UV–Vis absorption spectra after incubation with CT-DNA for 24 and 48 h respectively.

Compound	λ_max_ (nm)	Change in Absorbance	K_b_ × 10^6^ (M^−1^) Incubation 24 h	K_b_ × 10^6^ (M^−1^) Incubation 48 h
2	255.5	Hyperchromicity	4.0	2.0
9	258.5	Hypochromicity	66.7	22.2

**Table 2:** Free radical-scavenging activity of *N*,*N′*-dialkyl-9,9-biacridylidenes derivatives measured by DPPH and ABTS assay, respectively. Experiments were performed in triplicate. Results were expressed as IC_50_ ± standard deviation or TEAC values.

Analog	Radical Scavenging Activity (μM)	
	DPPH-Assay ^1^ IC_50_ ± sd (TEAC)	ABTS-Assay ^1^ IC_50_ ± sd (TEAC)
7	16.98 ± 0.88 (1.03)	32.32 ± 1.90 (3.68)
8	33.7 ± 2.1 (2.00)	9.33 ± 0.42 (1.07)
9	30.2 ± 1.17 (1.44)	21.45 ± 1.15 (1.14)
10	28.350 ± 3.1 (1.69)	16.88 ± 1.25 (1.92)
11	17.73 ± 0.53 (1.05)	12.43 ± 0.86 (1.42)
12	16.02 ± 0.56 (0.96)	24.1 ± 1.11 (1.52)
Caffeic acid	6.95 ± 0.46 (0.42)	8.67 ± 0.36 (0.99)
Trolox	16.76 ± 0.54 (1.0)	8.78 ± 0.29 (1.0)

^1^ Data are the mean value of three different determinations; trolox-equivalent antioxidant capacity (TEAC) values in parenthesis were calculated by dividing the mean of IC_50_ value of the analog through the IC_50_ value of trolox.

**Table 3:** IC_50_ Values (μΜ) for *N*-alkyl-acridone and *N*,*N′*-dialkyl-9,9-biacridylidene analogs treated with MCF-7 breast cancer cells for 24, 48, and 72 h incubation. As no total inhibition was observed for some derivatives the corresponding values could be given as relative IC_50_.

Analog	24 h	48 h	72 h
**1**	6.75	1.77	0.02
**2**	10.01	4.07	1.89
**3**	11.67	5.30	3.32
**4**	13.80	8.07	3.78
**5**	14.81	7.74	4.27
**6**	14.13	8.74	5.16
**7**	19.13	11.75	9.57
**8**	15.65	8.78	6.70
**9**	28.40	18.45	10.60
**10**	30.95	17.29	14.05
**11**	34.54	23.10	14.64
**12**	33.95	23.40	14.38

## Supplementary Materials

The following are available online at https://www.mdpi.com/2218-273X/9/5/177/s1, Figure S1: Topoisomerase-I mediated DNA relaxation assay of control acridone and *N*-alkylacridones **2**–**5** at pH 7.2; Figure S2: Topoisomerase-I mediated DNA relaxation assay of *N*,*N′*-dialkyl-9,9′-biacridylidenes **8, 9** and **10** at pH 7.2; Figure S3: Dose-dependent response of MCF-7 epithelial breast cancer cells to control acridone; Figure S4: Intracellular distribution of **9** and **11** after 24 h; Table S1: Free radical-scavenging activity of *N*,*N′*-dialkyl-9,9′-biacridylidenes calculated by the DPPH-assay; Table S2: Free radical-scavenging activity of *N*,*N′*-dialkyl-9,9′-biacridylidenes calculated by the APTS-assay.
