# Supplementary material for: Assessment of DNA Topoisomerase I Unwinding Activity, Radical Scavenging Capacity, and Inhibition of Breast Cancer Cell Viability of N-alkyl-acridones and N,N′-dialkyl-9,9′-biacridylidenes
Source: Biomolecules. 2019 May 8;9(5):177. doi: 10.3390/biom9050177 (PMC6572364; doi:10.3390/biom9050177)
Supplement: Supplementary file 1 [file biomolecules-09-00177-s001.pdf]

Supporting Information

Assessment of DNA topoisomerase I unwinding activity, radical scavenging capacity and inhibition of breast cancer cell viability of *N*-alkyl-acridones and *N,N'*-dialkyl-9,9'-biacridylidenes

Marios G. Krokidis, Zara Molphy, Eleni K. Efthimiadou, Marianna Kokoli, Smaragda-Maria Argyri, Irini Dousi, Annalisa Masi, Kyriakos Papadopoulos*, Andrew Kellett[2] and Chryssostomos Chatgilialoglu*

*CONTACT:
chrys@isof.cnr.it
k.papadopoulos@inn.demokritos.gr

List of Content:

1. Topoisomerase-I mediated DNA relaxation assay
2. Regression equations parameters of radical scavenging activity of DDPH and APTS assays
3. MTT assay of control acridone
4. Intracellular distribution of **9** and **11** after 24 h

1. Topoisomerase-I mediated DNA relaxation assay

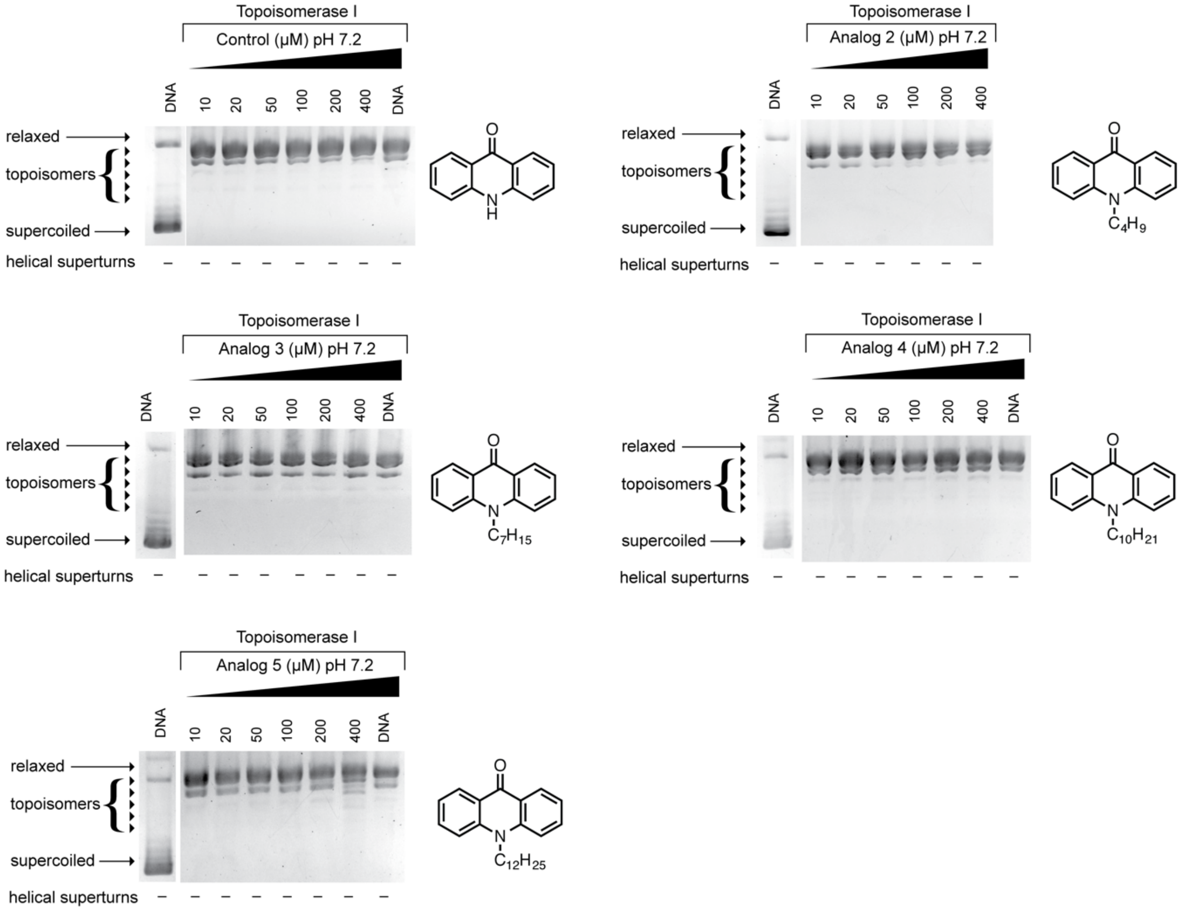

Figure S1: Topoisomerase-I mediated DNA relaxation assay in the presence of increasing concentrations (10-400 μM) of control acridone and *N*-alkylacridones **2-5** at pH 7.2.

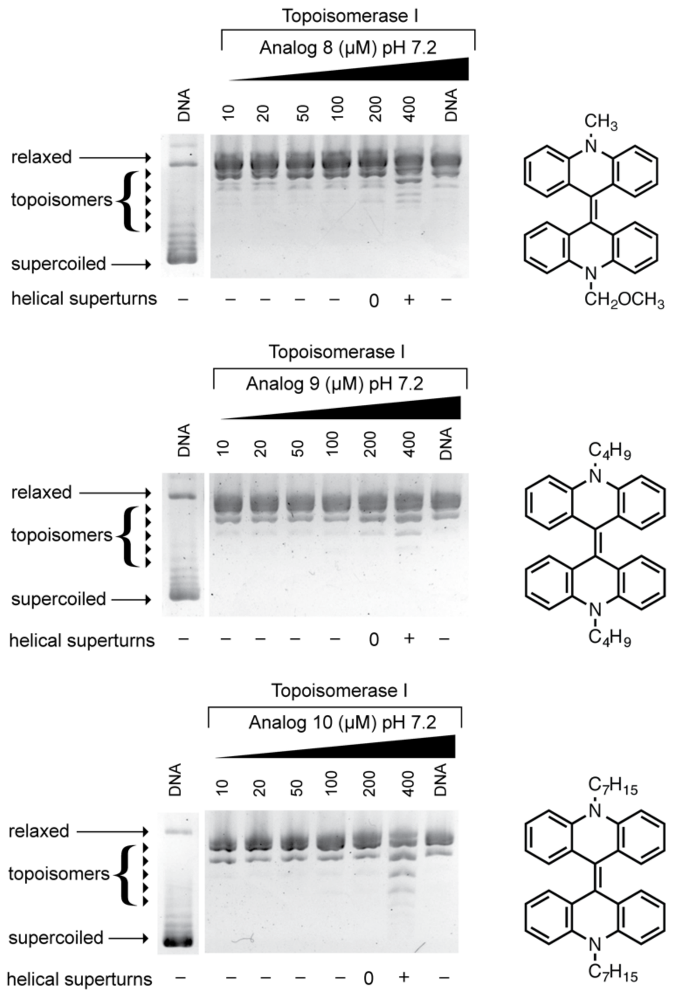

Figure S2: Topoisomerase-I mediated DNA relaxation assay in the presence of increasing concentrations (10-400 μM) of *N,N′*-dialkyl-9,9'-biacridylidenes **8, 9** and **10** at pH 7.2.

2. Regression equations parameters of radical scavenging activity of DDPH and APTS assays

Table S1: Free radical-scavenging activity of *N,N'*-dialkyl-9,9'-biacridylidenes derivatives along with their corresponding regression equations parameters calculated by the DPPH-assay. Results were expressed as IC_{50}± standard deviation or TEAC values.

**Analog**	**Lineal range (µM)**	**Slope**	**correlation coefficient (r^2)**	**Radical scavenging activity** IC_{50} ± sd (TEAC)
**7**	5.0-25	2.7975	0.9943	16.98±0.88 (1.03)
**8**	10-50	2.6325	0.9824	33.7±2.1 (2.00)
**9**	10-50	2.346	0.9711	28.350 ± 3.1(1.69)
**10**	10-50	1.587	0.9739	28.350 ± 3.1 (1.69)
**11**	5.0-20	2.7361	0.9643	17.73±0.53 (1.05)
**12**	5.0-20	3.0161	0.956	16.02±0.56 (0.96)
Caffeic acid	1.0-10.0	6.7494	0.9835	6.95±0.46 (0.42)
Trolox	2.5-50.0	2.8673	0.9781	16.76±0.54 (1.0)

Equations were calculated using five different concentrations assayed in triplicate. All equations followed a linear regression model. TEAC-values were calculated by dividing the IC_{50} value of each analog through the IC_{50} value of trolox.

Table S2: Free radical-scavenging activity of *N,N'*-dialkyl-9,9'-biacridylidenes derivatives along with their corresponding regression equations parameters calculated by the APTS-assay. Results were expressed as IC_{50}± standard deviation or TEAC values.

**Antioxidant**	**Linear range (µM)**	**Slope**	**Correlation coefficients (r^2)**	**Radical scavenging activity** IC_{50} ± sd (TEAC)
**7**	5.0-50	1.3787	0.9766	32.32±1.90 (3.68)
**8**	10-50	2.2452	0.9824	9.33±0.42 (1.07)
**9**	10-50	2.845	0.9722	21.45±1.15 (1.14)
**10**	10-50	2.7576	0.9725	16.88±1.25 (1.92)
**11**	5.0-25	3.5243	0.9788	12.43±0.86 (1.42)
**12**	5.0-50	1.7882	0.9778	24.1±1.11 (1.52)
Caffeic acid	2.5-15	5.5325	0.9923	8.67±0.36 (0.99)
Trolox	2.5-12.5	5.8934	0.9961	8.78±0.29 (1.0)

Equations were calculated using five different concentrations assayed in triplicate. All equations followed a linear regression model. TEAC-values were calculated by dividing the IC_{50} value of each analog through the IC_{50} value of trolox.

3. MTT assay of control acridone

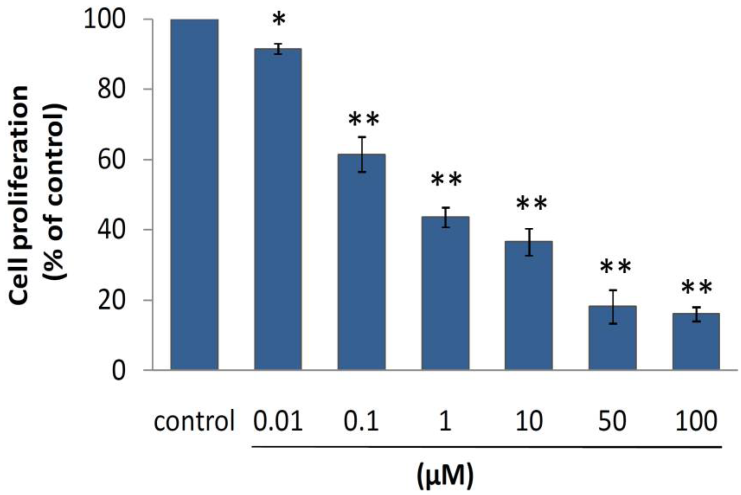

Figure S3: Dose-dependent response of MCF-7 epithelial breast cancer cells to acridone (0.01 to 100 µM) for 24 h in serum containing medium. The results are presented as percentage of growth in respect to control cells. Each point represents the mean ± standard deviation from experiments in triplicate. Asterisks mark the statistically significant levels using the Student t-test: *$p<0.05$, **$p<0.01$, respectively, as compared to control.

4. Intracellular distribution of 9 and 11 after 24 h

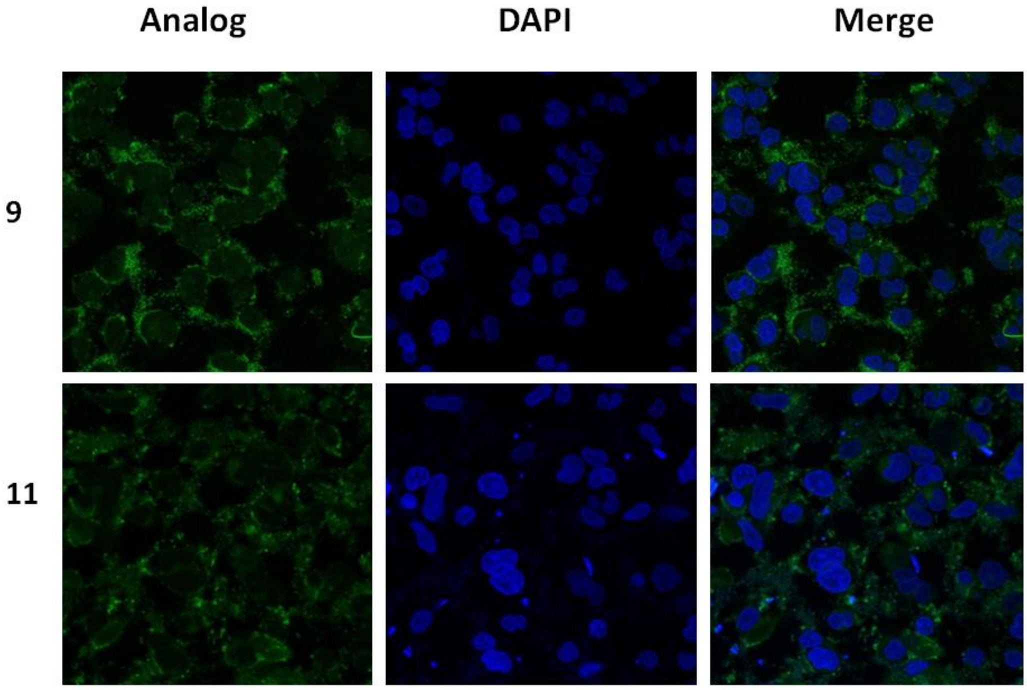

Figure S4: Determination of intracellular distribution of analogs **9** and **11** in MCF-7 breast cancer cells. Cells were treated with 1 μM of each derivative and after 24 h incubation cells were imaged by confocal microscopy. Blue is DAPI (4′,6-diamidino-2-phenylindole) nuclear stain.
